# Endovascular treatment of intracranial internal carotid artery bifurcation region aneurysms

**DOI:** 10.3389/fneur.2024.1344388

**Published:** 2024-03-28

**Authors:** Xu Liu, Yunbao Guo, Kun Zhang, Jinlu Yu

**Affiliations:** ^1^Department of Neurosurgery, First Hospital of Jilin University, Changchun, China; ^2^Department of Cerebrovascular Disease, Henan Provincial People’s Hospital of Zhengzhou University, Zhengzhou, China

**Keywords:** internal carotid artery bifurcation, aneurysm, endovascular treatment, prognosis, complication

## Abstract

Intracranial internal carotid artery (ICA) bifurcation region aneurysms are uncommon. When treatment is necessary for ICA, endovascular treatment (EVT) can be a useful option. Due to the complexity of these aneurysms and the variability of EVT techniques, EVT for ICA bifurcation aneurysms is challenging. Currently, it is necessary to perform a review to explore this issue further. In this review, the following issues were discussed: the anatomy of the ICA bifurcation region; the classification, natural history and EVT status of ICA bifurcation region aneurysms; the technique used for identifying ICA bifurcation region aneurysms; and the prognosis and complications of EVT for ICA bifurcation region aneurysms. According to the review and our experience, traditional coiling is currently the preferred therapy for ICA bifurcation region aneurysms. In addition, in select cases, new devices, such as flow diverters and Woven EndoBridge devices, can also be used to treat ICA bifurcation region aneurysms. Generally, EVT is an alternative treatment option for ICA bifurcation region aneurysms.

## 1 Introduction

The intracranial internal carotid artery (ICA) bifurcation region is centered on the ICA terminus, and this region can involve the distal ICA segment beyond the anterior choroidal artery (AchA), middle cerebral artery (MCA) and anterior cerebral artery (ACA) origins (1, 2). Aneurysms can occur in this region because increased hemodynamic stress at the level of arterial bifurcations has been linked to the development of aneurysms or aneurysmal rupture (3, 4). ICA bifurcation region aneurysms have a relatively high incidence in young patients, accounting for 2%–9% of all intracranial aneurysms (5, 6).

Ruptured or daughter-sac/multilobed, large or giant, growing ICA bifurcation region aneurysms may need treatment, including open surgery and endovascular treatment (EVT) (7–10). Currently, EVT, including traditional coiling, flow diverter (FD) and Woven EndoBridge device (WEB) (MicroVention, Tustin, California, United States) deployment, has been used for the treatment of ICA bifurcation region aneurysms (11, 12). However, EVT is challenging due to the unfavorable morphologic features of ICA bifurcation aneurysms. Due to the complexity of EVT and insufficient understanding of ICA bifurcation region aneurysms, it was necessary to perform a review to explore this issue further.

## 2 Anatomy of the ICA bifurcation region

**Figure 1:**
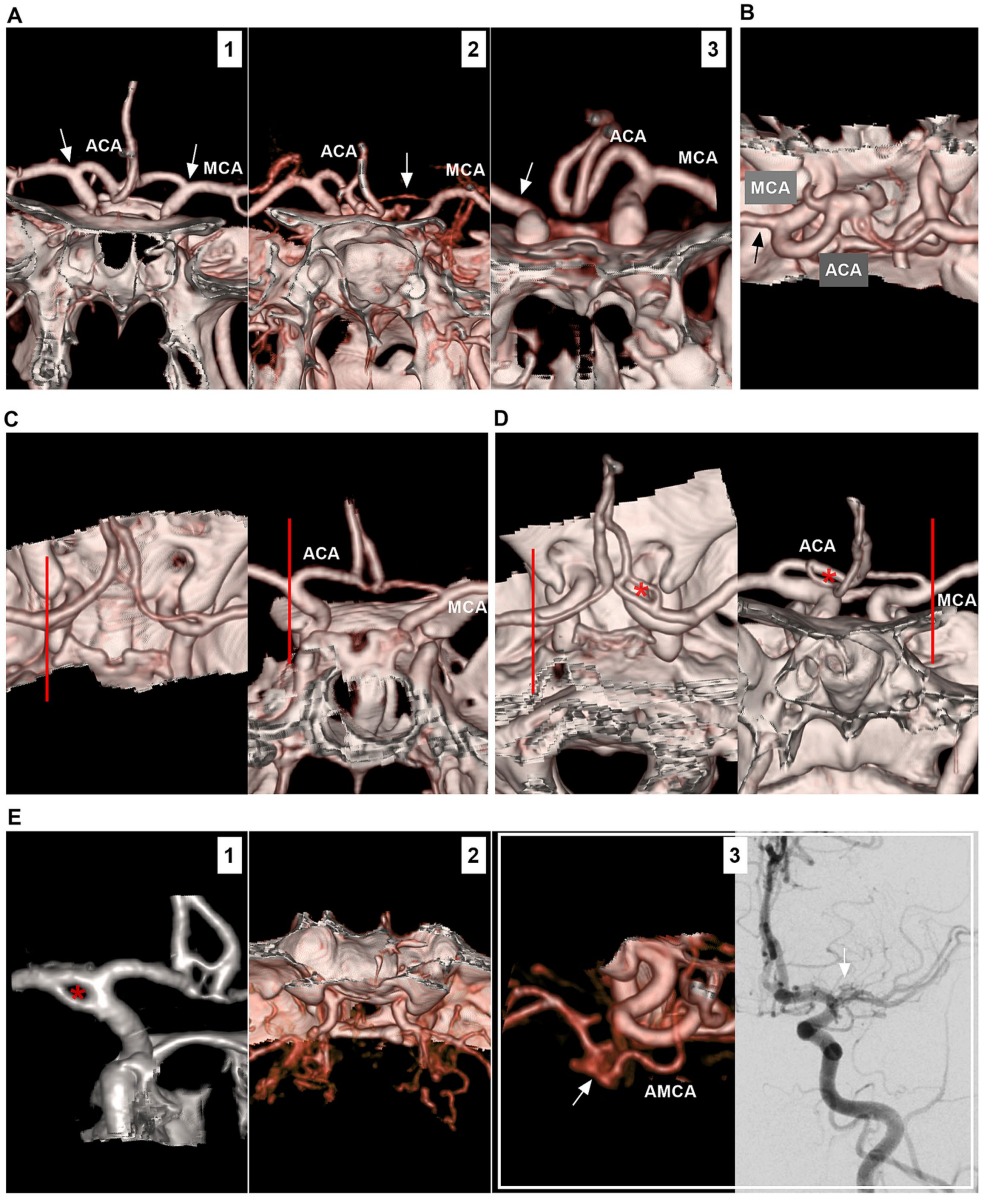
Anatomy and anomaly of the ICA bifurcation region. **(A)** Number 1 panel: CTA showing symmetrical ICA bifurcations (arrows), the ACA was well-developed. Number 2 panel: CTA showing asymmetrical ICA bifurcations (arrows), and the unilateral ACA (arrow) was hyperplastic. Number 3 panel: CTA showing that unilateral ACA (arrow) was absent, and there was no true ICA bifurcation (arrow). **(B)** CTA showing that, in the ICA bifurcation region, the ACA was thicker than the MCA (arrow). **(C)** In the left and right panels (different views), CTA showing the ICA bifurcation located at the level of anterior clinoid process (lines). **(D)** In the left and right panels (different views), CTA showing the ICA bifurcation located outside anterior clinoid process (lines); the fenestration (asterisks) on the ACA can be seen. **(E)** Number 1 panel: CTA showing a fenestration (asterisk) at the ICA bifurcation. Number 2 panel: CTA showing moyamoya disease involving bilateral ICA bifurcations. Number 3 panel: CTA (left) showing that the twig-like MCA (arrow); the AMCA supplied the twig-like MCA; DSA (right) showing the twig-like MCA (arrow). ACA, anterior cerebral artery; AMCA, accessory middle cerebral artery; CTA, computed tomography angiography; DSA, digital subtraction angiography; ICA, internal carotid artery; MCA, middle cerebral artery.

The ICA bifurcation region is located lateral to the optic chiasm and below the anterior perforated substance at the medial end of the sylvian fissure. The ICA bifurcation has a complex anatomy, and its location may project laterally or posteriorly. The ACA may be hyperplastic, hypoplastic or absent; in addition, fenestration, moyamoya disease and the twig-like MCA may involve the ICA bifurcation (Figure 1).

**Figure 2:**
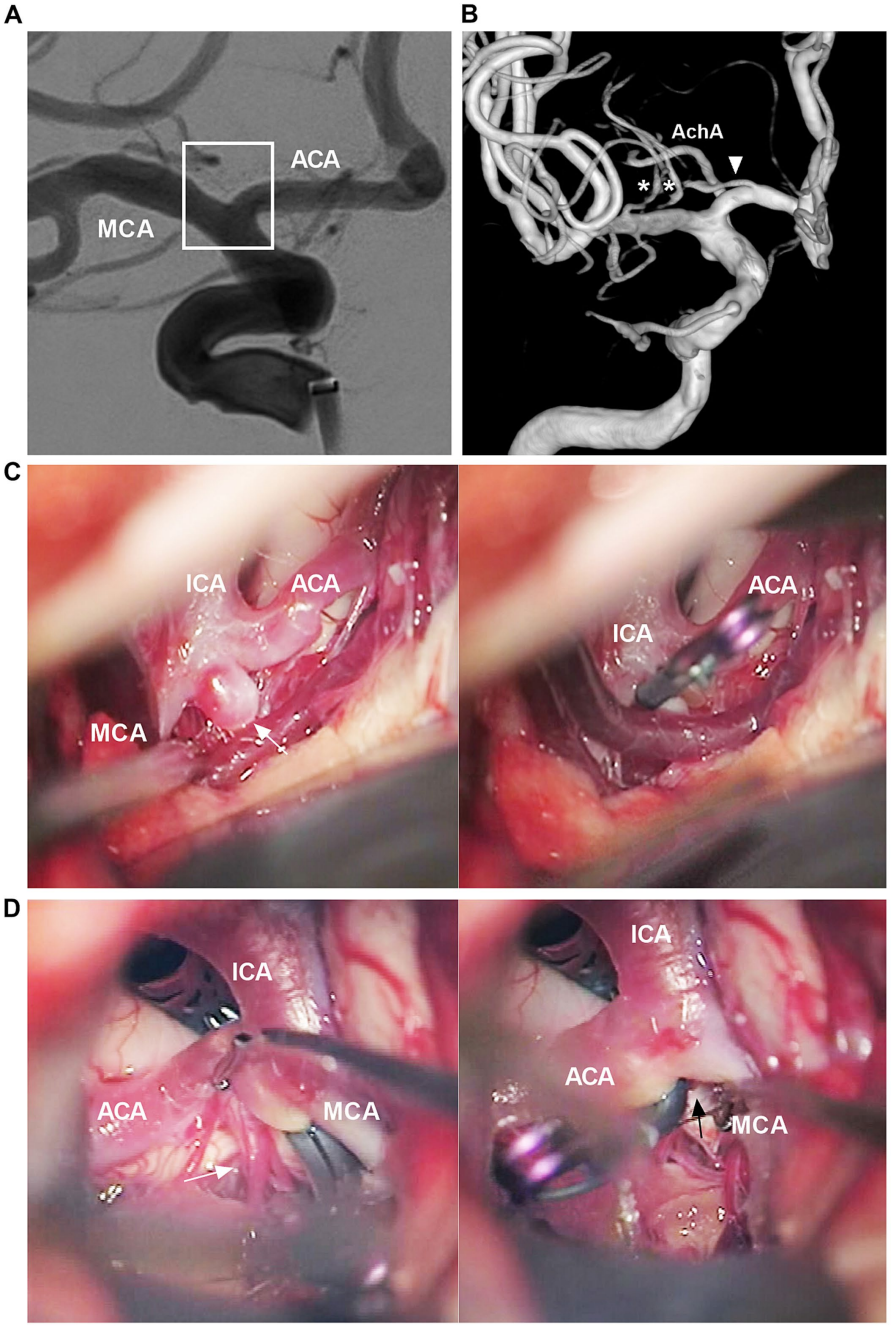
Angiographic and intraoperative findings of the ICA bifurcation region. **(A)** DSA showing no visible perforating arteries from the ICA bifurcation region (frame). **(B)** Three-dimensional DSA showing that the recurrent artery of Heubner (arrowhead) and lateral lenticulostriate arteries (asterisks) course across the ICA bifurcation region. **(C)** Intraoperative image showing that, in the left panel, no major perforating arteries can be seen from the ICA bifurcation region. The ICA bifurcation aneurysm was clipped (arrow in the right panel). **(D)** Intraoperative image after clipping the ICA bifurcation aneurysm showing, in left panel, a group of perforating arteries (arrow) can be seen from the A1 origin, in right panel, no major perforating arteries can be seen from the ICA bifurcation region (arrow). ACA, anterior cerebral artery; AchA, anterior choroidal artery; DSA, digital subtraction angiography; ICA, internal carotid artery; MCA, middle cerebral artery.

The ICA bifurcation region may be devoid of larger perforating arteries. Usually, only 3–5 perforating arteries <0.5 mm in diameter arise from the midportion of the ICA bifurcation superiorly to the medial portion of the anterior perforated substance; these perforating arteries are often difficult to locate via digital subtraction angiography (Figure 2A) (13). However, perforating branches of adjacent cerebral arteries can cross the ICA bifurcation region, including the ACA, MCA, AchA and recurrent artery of Heubner (Figure 2B) (14, 15). Intraoperative images obtained during clipping of the aneurysm in the ICA bifurcation region also confirmed these angiographic findings (Figures 2C,D).

## 3 Classifications of ICA bifurcation region aneurysms

### 3.1 Location

Broadly speaking, ICA bifurcation region aneurysms can include those beyond the AchA involving the ICA terminus and those at the ICA bifurcation, ICA-A1 junction or ICA-M1 junction (7). In a narrow sense, ICA bifurcation aneurysms refer only to those at the ICA apex. Those that arose purely from the proximal A1 or M1 were excluded (16).

### 3.2 Saccular or dissecting

**Figure 3:**
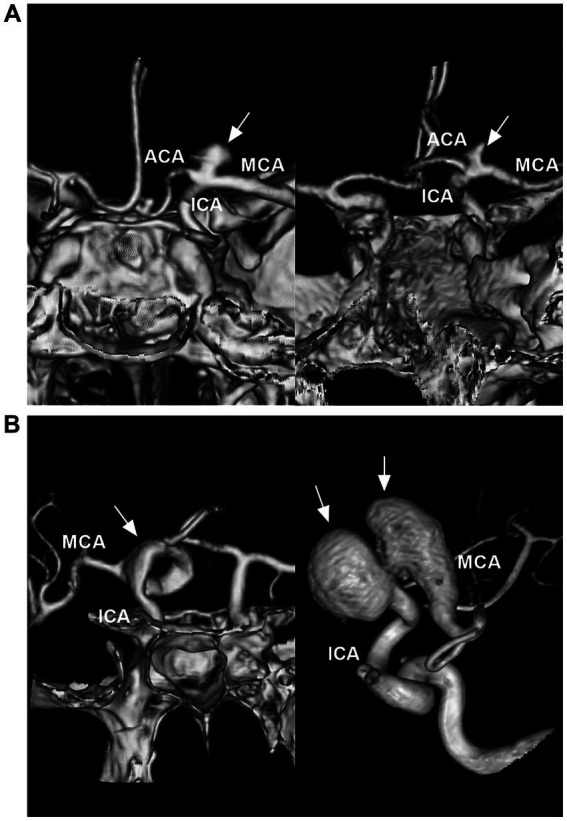
Classifications of ICA bifurcation region aneurysms. **(A)** Left panel: CTA showing a symmetric type of saccular aneurysm (arrow) that is on the highest point of the ICA. Right panel: CTA showing a M1 predominant type of saccular aneurysm (arrow). **(B)** Left panel: CTA showing a serpentine aneurysm (arrow) involving the ICA terminus. Right panel: Three-dimensional DSA showing a tandem of dissecting aneurysms (arrows) involving the ICA terminus and MCA origin. ACA, anterior cerebral artery; CTA, computed tomography angiography; DSA, digital subtraction angiography; ICA, internal carotid artery; MCA, middle cerebral artery.

Saccular aneurysms settled on the ICA bifurcation and had a neck (Figure 3A). They can project superiorly, posteriorly, anteriorly, laterally or medially, based on the aneurysmal dome relative to the distal ICA axis (17, 18). Saccular aneurysms can be categorized as symmetric and centered on the ICA bifurcation, with A1 or M1 being the predominant type. Symmetric-type aneurysms account for 50% of aneurysms, followed by the M1-and A1-predominant types (7). ICA bifurcation region dissecting aneurysms had no neck and presented with fusiform dilation involving the ICA terminus (Figure 3B) (19).

### 3.3 Other classifications

According to the International Subarachnoid Aneurysm Trial (ISAT) (20), ICA bifurcation region aneurysms can be divided into small (<7 mm), medium (7–12 mm), large (>12–25 mm) or giant (>25 mm) lesions. Giant aneurysms are uncommon and account for 7% of all these lesions (19, 21, 22). ICA bifurcation region aneurysms can be divided into wide-necked (neck width ≥4.0 mm or dome-to-neck ratio <2.0) and narrow-necked (neck width <4 mm, dome-to neck ratio >2) aneurysms (23). In addition, ICA bifurcation region aneurysms can be spontaneous, traumatic or secondary to radiation (24, 25). They can be bilateral mirror-like or can involve multiple lesions associated with other aneurysms (even up to 50% in patients with ICA bifurcation region aneurysms) (6, 8, 19, 26–28).

## 4 Natural history of ICA bifurcation region aneurysm

Rupture of an intracranial aneurysm is a catastrophic event. Without access to treatment, the fatality rate is 50% in the first 30 days, including for ICA bifurcation region aneurysms. Rebleeding is the most imminent danger (29). Therefore, ruptured ICA bifurcation region aneurysms have a malignant natural history, aggressive treatment can be recommended.

According to the ISAT (20) and the Japanese Study on Unruptured Aneurysms (30), there is no direct information on the natural history of unruptured ICA bifurcation region aneurysms. These aneurysms are often mixed into intracranial ICA aneurysms for study (31). In Rinkel’s et al. (32) review of intracranial ICA aneurysms, including ICA bifurcations, during 2,449 patient-years, the overall risk of rupture per patient-year was 1.2%, and the risk of rupture per patient-year was 0.7% for those with a size ≤10 mm and 4% for those with a size >10 mm. In the natural course of unruptured cerebral aneurysms in a Japanese cohort (30), of 1,245 intracranial ICA aneurysms, including those of the ICA bifurcation, the annual rupture rates were 0.14%, 0, 1.19, 1.07, and 10.61% for aneurysms 3–4 mm, 5–6 mm, 7–9 mm, 10–24 mm, and ≥25 mm in length, respectively. Because some paraophthalmic aneurysms with a benign history were included in above studies, unruptured ICA bifurcation region aneurysms may have a greater risk of rupture than whole intracranial ICA segment aneurysms.

Currently, it is accepted that aneurysms in the smaller ICA bifurcation region may have a much lower risk of growth and rupture (33). Because the intracranial ICA bifurcation region is considered a terminal vessel, similar to other intracranial bifurcations, this region is associated with special hemodynamic stress and high wall shear stress (17, 34). When ICA bifurcation region aneurysms are associated with aneurysms in other locations or are multilobed, daughter sac, large or giant, or growing, their natural history may be poor (20, 30, 35–37). Therefore, for these lesions, aggressive treatment can be considered.

## 5 Open surgery and EVT status

In the current EVT era, an increasing number of aneurysms are being treated through EVT. As a result, microsurgical clipping is often reserved for complex ICA bifurcation region aneurysms, especially for those that need bypass (15, 16, 38–42). In addition, open surgery may be helpful for treating ICA bifurcation region aneurysms with intracerebral hematomas that require decompression (42). However, deep retraction is needed for surgical exposure and clipping of ICA bifurcation region aneurysms to achieve good exposure, the high position of the aneurysm with respect to the skull base increases the difficulty of microsurgery, and the attachment of the aneurysm dome to the surrounding brain parenchyma and rich perforators in this region also increases the difficulty of microsurgery (8).

**Figure 4:**
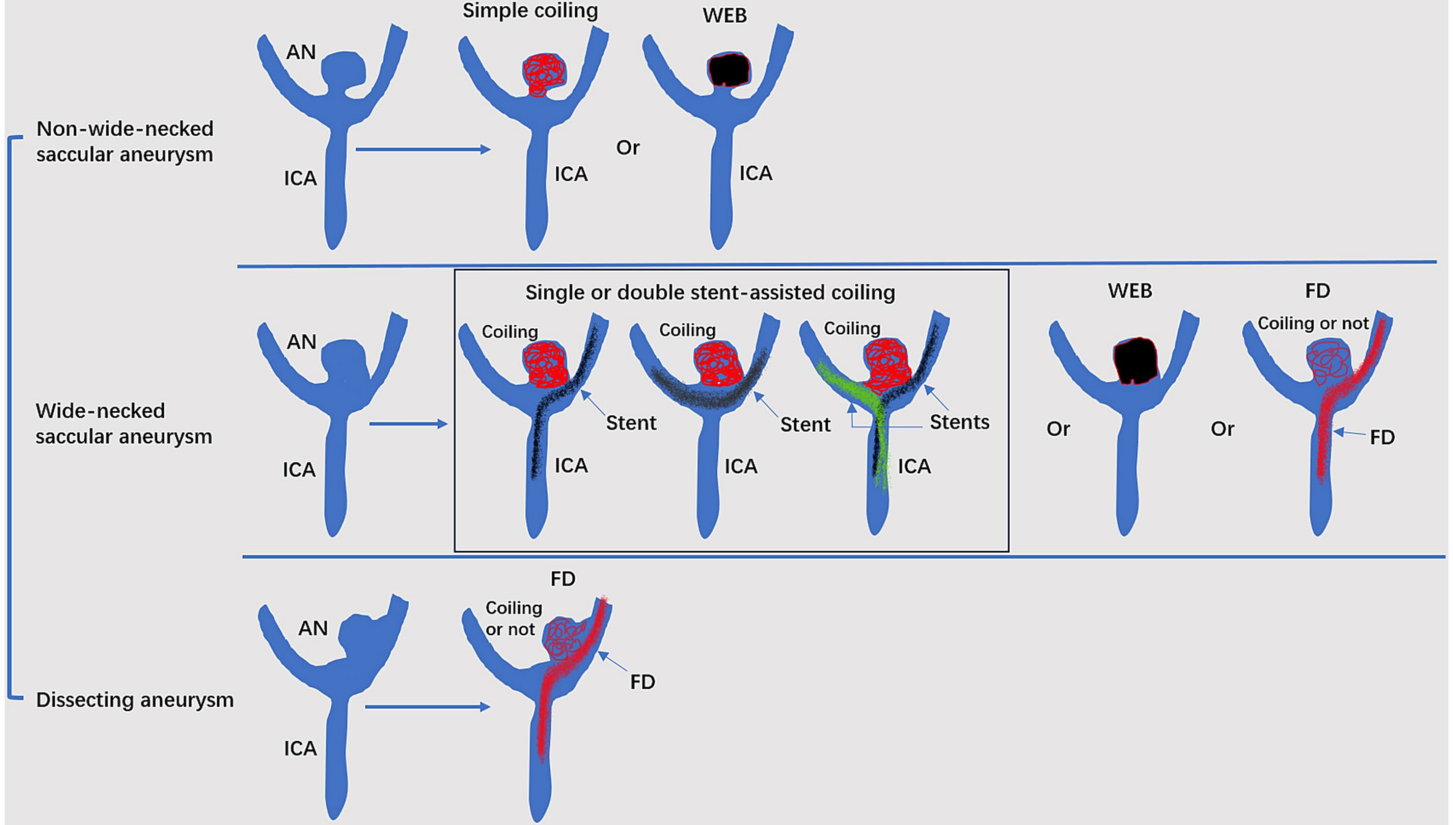
Mode pattern of the EVT of ICA bifurcation region aneurysms. ACA, anterior cerebral artery; AN, aneurysms; EVT, endovascular treatment; FD, flow diverter; ICA, internal carotid artery; MCA, middle cerebral artery; WEB, Woven EndoBridge device.

Undeniably, EVT can now be used for intracranial aneurysms based on robust randomized controlled trial data. EVT, including traditional coiling, FD and WEB device deployment, can be a useful option for ICA bifurcation region aneurysms (Figure 4). However, EVT is challenging due to the complexity of aneurysms.

## 6 Various EVT techniques

### 6.1 Traditional coiling

**Figure 5:**
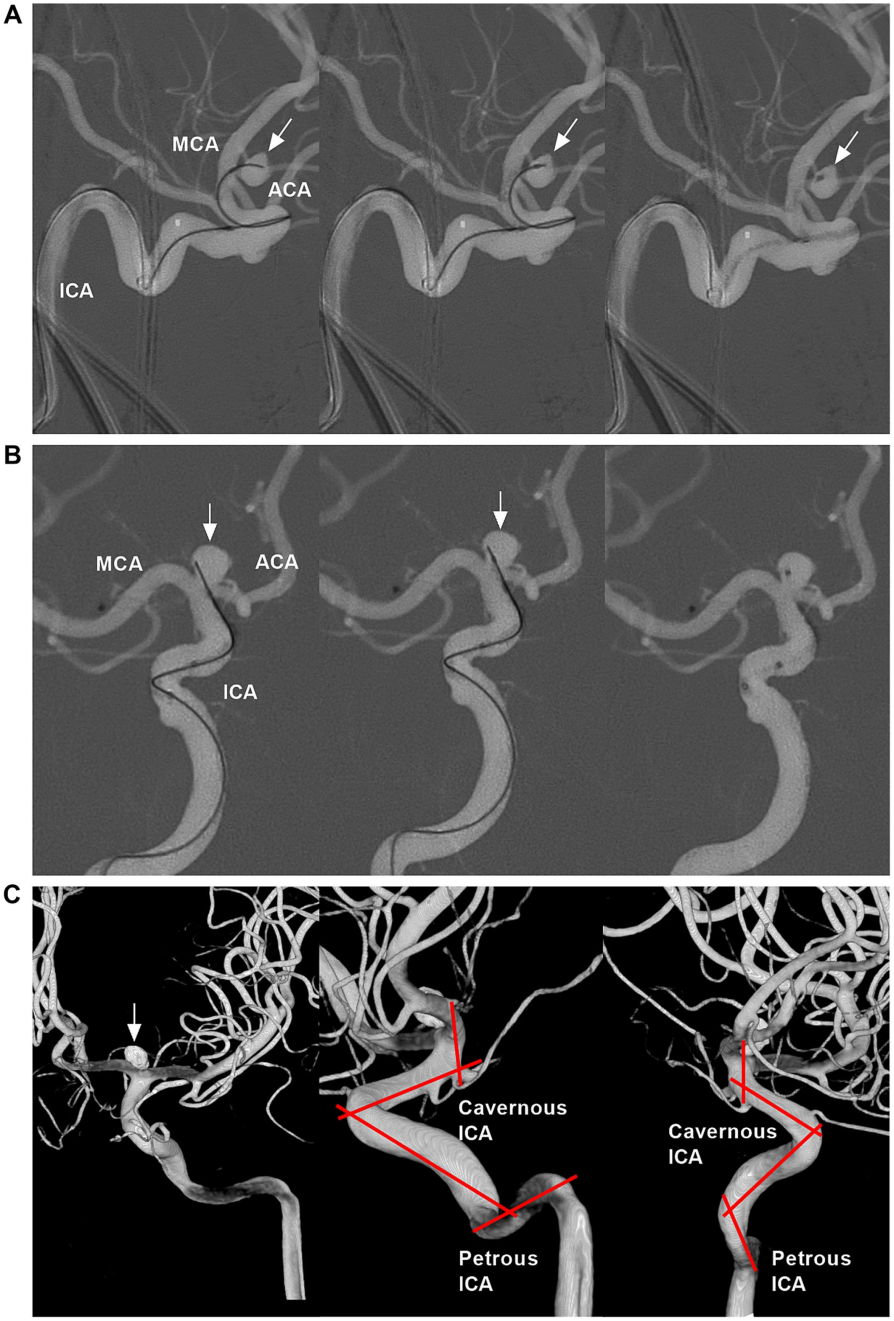
Catheterization for the ICA bifurcation aneurysms and tortuosity of cavernous and petrous ICA segments. **(A)** Roadmap DSA showing successful catheterization for ICA bifurcation aneurysm (arrows) using a microcatheter with “C” shape tip. **(B)** Roadmap DSA showing successful catheterization for ICA bifurcation aneurysm (arrows) using a microcatheter with straight tip. **(C)** Three-dimensional DSA showing an ICA bifurcation aneurysm (arrow) that was coiled; the zigzag lines indicated the tortuosity of cavernous and petrous ICA segments. ACA, anterior cerebral artery; DSA, digital subtraction angiography; ICA, internal carotid artery; MCA, middle cerebral artery.

Currently, traditional coiling is still the preferred method for treating ICA bifurcation aneurysms. Due to the various directions and deviations, catheterization is often difficult. For aneurysms with superior projections and those with narrow necks, catheterization may be easy (Figures 5A,B) (17). Microcatheters with various tip shapes can be used to access ICA bifurcation saccular aneurysms based on the axis of aneurysm and ICA, and the tortuosity of the petrous and cavernous ICA (Figure 5C). In a study by Lee et al. (16), during the catheterization of ICA region saccular aneurysms, a steam-shaped “S” microcatheter was most commonly used, followed by a preshaped 45-degree, 90-degree and straight tip.

**Figure 6:**
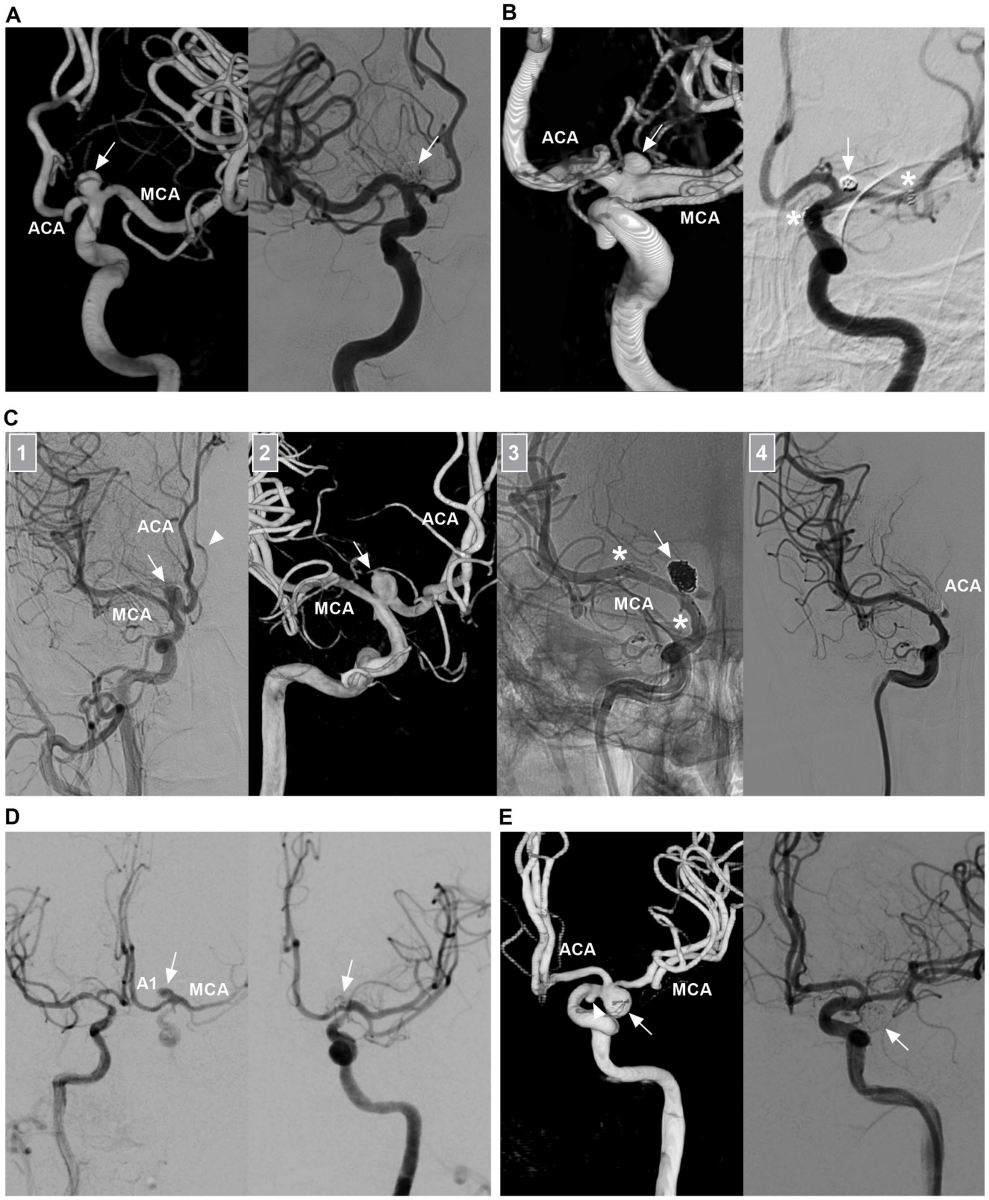
Traditional EVT of ICA bifurcation aneurysms. **(A)** Left panel: Three-dimensional DSA showing a symmetric type ICA bifurcation aneurysm (arrow). Right panel: DSA showing that the aneurysm (arrow) was coiled. **(B)** Left panel: Three-dimensional DSA showing a M1 predominant type ICA bifurcation aneurysm (arrow) with a wide neck. Right panel: 6 months follow-up DSA showing that the aneurysm (arrow) was coiled under the assistance of stenting from the MCA to ICA; the asterisks indicated the tips of the stent. **(C)** Number 1 panel: DSA showing an A1 predominant type ICA bifurcation aneurysm (arrow) with a wide neck, the arrowhead indicated the contralateral ACA. Number 2 panel: Three-dimensional DSA showing the wide-necked aneurysm (arrow). Number 3 panel: Unsubtracted DSA showing that the aneurysm (arrow) was coiled under the assistance of stenting (asterisks) from the MCA to ICA. Number 4 panel: DSA showing that the ipsilateral ACA was occluded. Postoperatively, the patient had no symptom due to ACA occlusion. **(D)** DSA showing that an ICA bifurcation aneurysm (arrow) can be seen via the contralateral A1 and AcomA, after the ipsilateral ICA was compressed. Right panel: DSA showing that the aneurysm (arrow) was coiled. **(E)** Left panel: Three-dimensional DSA showing an ICA bifurcation aneurysm (arrow) pointing inferiorly and a small AchA aneurysm (arrowhead). Right panel: DSA showing that the ICA region aneurysm (arrow) was coiled, the AchA aneurysm was untreated. 3D, three-dimensional; ACA, anterior cerebral artery; AchA, anterior choroidal artery; AcomA, anterior communicating artery; A1, first segment of the ACA; DSA, digital subtracted angiography; ICA, internal carotid artery; MCA, middle cerebral artery.

When a stable coil frame is not produced with a single microcatheter, supplemental maneuvers can be used, such as double microcatheters, balloon remodeling coiling and single or double “Y” configuration stent assisting coiling (Figure 6) (6, 34, 43–45). When the contralateral A1 and anterior communicating artery (AcomA) are sufficient, the transcirculation stenting technique can be used. In this technique, a stent can be advanced through the AcomA from the contralateral A1 and placed horizontally across the aneurysm neck; then, the aneurysm can be coiled by an ipsilateral microcatheter (46–49). In addition, when the contralateral A1 segment and Acoma were patent, proximal ACA occlusion with an aneurysm and reversal of flow in the ipsilateral A1 segment may be feasible (Figure 6C).

### 6.2 WEB deployment

The WEB device is designed for wide-necked bifurcation aneurysms and is typically used at four typical locations: the MCA bifurcation, AcomA, basilar apex and ICA apex (12, 50). After deployment in the aneurysm sac, the device provides immediate flow disruption, leading to aneurysm thrombosis and endothelialization across the aneurysm neck over time. Because the device leaves the parent artery unaffected, long-term antiaggregant therapy is not necessary (51). Several previous landmark studies, including WEBCAST, WEBCAST-2, WEB-IT, and the French Observatory, have confirmed the safety and efficacy of the WEB device (52–56).

**Figure 7:**
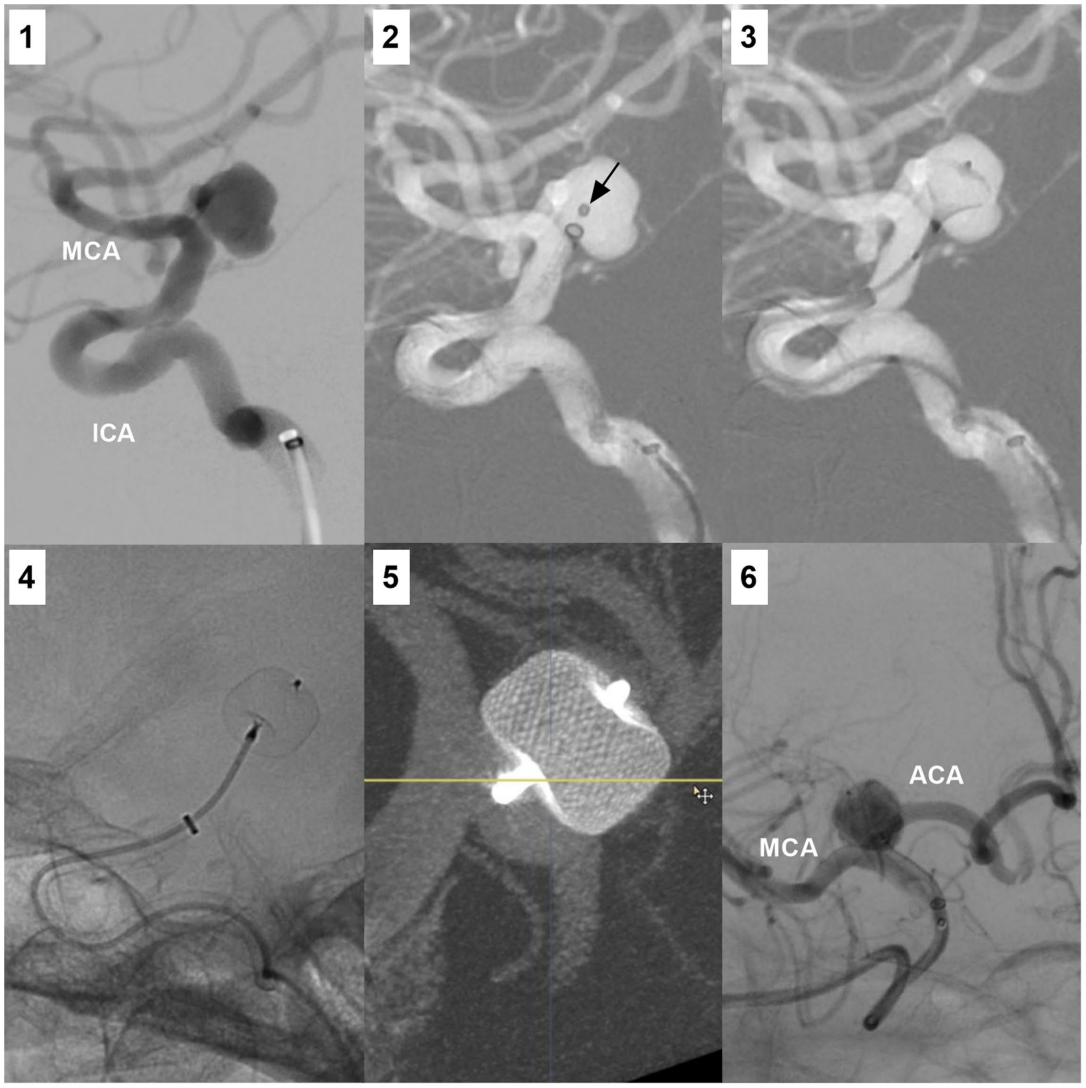
WEB device deployment for an ICA bifurcation aneurysm. Number 1 panel: DSA showing an ICA bifurcation aneurysm. Number 2 panel: Roadmap DSA showing the microcatheter (arrow) in the aneurysm. Number 3 panel: Roadmap DSA showing the partially open WEB device in the aneurysm. Number 4 panel: X-ray image showing the open WEB device. Number 5 panel: Vascular reconstructive DSA showing the WEB device in the aneurysm. Number 6 panel was the postoperative DSA. ACA, anterior cerebral artery; DSA, digital subtraction angiography; ICA, internal carotid artery; MCA, middle cerebral artery; WEB, Woven EndoBridge device.

The WEB device can be used for ICA bifurcation aneurysms. After deployment, the aneurysm dome and ruptured bleb can be protected from rerupture despite residual flow into the aneurysm base (Figure 7) (57–59). When deployed, the WEB device is typically oversized by 1 mm in width and undersized in height by 1 mm (+1/−1 rule) (60, 61). Oversizing the width was predictive of better occlusion (11, 61). However, WEB deployment has several limitations. WEB devices for large aneurysms >10 mm in size were unavailable. Only aneurysms 2–10 mm can be treated by a WEB device (60). In addition, the WEB device cannot be used for thrombosed aneurysms or pseudoaneurysms (61).

### 6.3 FD deployment

**Figure 8:**
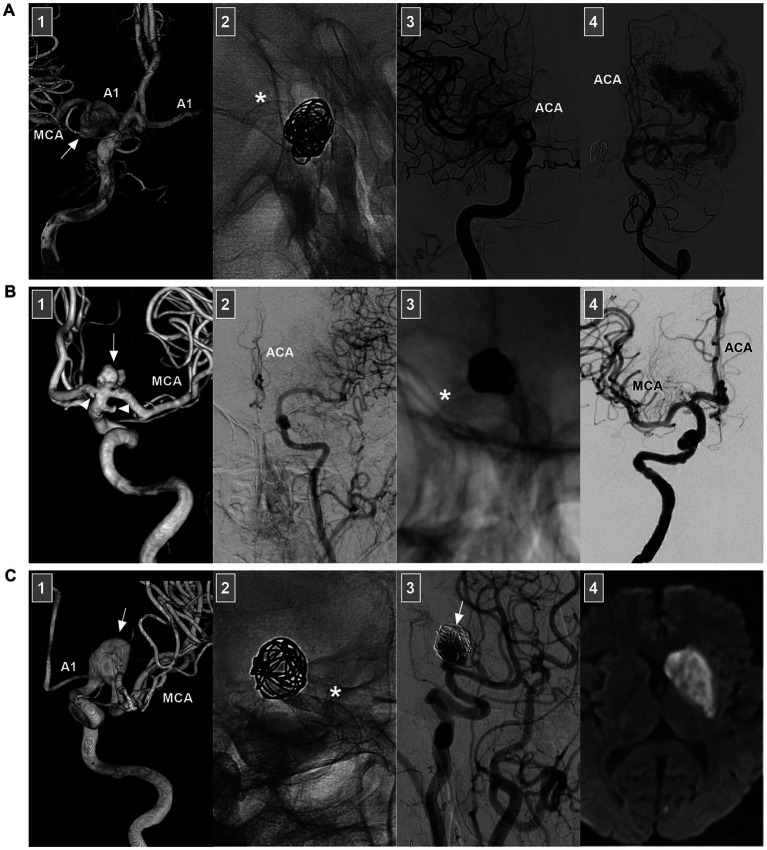
FD deployment of ICA bifurcation region aneurysms. **(A)** Number 1 panel: Three-dimensional DSA of the ICA showing an unruptured fusiform dissecting aneurysm (arrow) beyond the AchA involving the ICA terminus, the AcomA was patent, and the contralateral A1 can be seen. Number 2 panel: X-ray image showing the coils and FD deployment (asterisk). Number 3 panel: DSA of the ICA showing that the ipsilateral ACA was occluded. Number 4 panel: DSA of VA showing the arteriovenous malformation opposite the aneurysm; the ACA can be seen. Postoperatively, the patient had no symptom due to ACA occlusion. **(B)** Number 1 panel: Three-dimensional DSA showing an unruptured saccular aneurysm (arrow) at the ICA terminus; the associated A1 origin and AchA aneurysms (arrowheads) were found. Number 2 panel: DSA of the contralateral ICA showing the hypoplastic proximal ACA. Number 3 panel: X-ray image showing the coils and FD deployment (asterisk). Number 4 panel: Postoperative DSA showing that the ACA and MCA were patent. **(C)** Number 1 panel: Three-dimensional DSA showing an unruptured fusiform dissecting aneurysm (arrow) at M1 origin involving the ICA terminus. Number 2 panel: X-ray image showing the coils and FD deployment (asterisk). Number 3 panel: 6 months follow-up DSA of the ICA showing that the aneurysm (arrow) was occluded partially. Number 4 panel: After the transition to single antiplatelet agent, the patient suffered acute infarction of the caudate nucleus on magnetic resonance imaging. ACA, anterior cerebral artery; A1, first segment of the ACA; AchA, anterior choroidal artery; AcomA, anterior communicating artery; DSA, digital subtracted angiography; ICA, internal carotid artery; MCA, middle cerebral artery; VA, vertebral artery.

FDs with high mental coverage can restrain blood flow into aneurysms and promote thrombosis, resulting in subsequent endothelialization over the aneurysm neck (62). FDs are designed with a low porosity that diminishes flow to the treated aneurysm but still allows flow to the side branches or perforators of the parent vessel (63–65). Highly selective saccular and dissecting aneurysms of the ICA bifurcation region represent potential FD applications, such as aneurysms with a large irregular morphology or potential risk of recanalization after coiling (Figures 8A,B) (2). FD deployment should mainly be limited to unruptured aneurysms and reserved only as a last resort for ruptured aneurysms.

When using FD to treat ICA bifurcation region aneurysms, FD deployment may be deployed from the MCA to the ICA or from the ACA to the ICA. Rarely, horizontal FD deployment through the contralateral ICA can be performed (66). Due to flow competition, the pressure gradient of ACA or MCA covered by FD decreases. The involved brain territory begins to receive blood flow from collaterals to reconstitute blood flow. Because the MCA is a terminal vessel with no direct collateral circulation, FD deployment from the ACA to the ICA to cover the MCA origin should be used cautiously to avoid ischemia.

When there is no well-developed contralateral A1 or AcomA, the ipsilateral ACA is also a terminal vessel, and FD deployment from the MCA to the ICA to cover the ACA should also be used cautiously. When there is a well-developed contralateral A1 and AcomA, direct and sufficient blood flow can be ensured immediately from the collateral ACA via the AcomA. The fate of ACA may be patent, flow-reduced or occluded (1, 67, 68). After FD deployment from the MCA to the ICA, although the ICA bifurcation aneurysm is not covered too much, the aneurysm can be occluded due to flow modification.

### 6.4 New devices

In addition to the WEB device, other intrasaccular flow disruptors, such as the Cerus Endovascular Neqstent device and Contour Neurovascular System (CNS) (Cerus Endovascular, Fremont, CA, United States), the Luna/Artisse embolization system (Medtronic, Irvine, CA, United States), and the Medina Embolic Device (MED) (Medtronic, Irvine, CA, United States), can be used in selective ICA bifurcation region aneurysms (69–72). Stent-like devices, including the pCONus device (Phenox GmbH, Bochum, Germany), the eCLIP device (Evasc Medical Systems, Vancouver, Canada), and the PulseRider device (Cerenovus, Irvine, CA, United States), can be used for the treatment of ICA bifurcation region aneurysms (73). However, additional evidence is needed for the deployment of these new devices.

## 7 Prognosis and complications

### 7.1 Prognosis

For EVT for intracranial ICA bifurcation region aneurysms, a good clinical outcome can be defined as a modified Rankin scale (mRS) score ≤2 or a Glasgow Outcome Scale (GOS) score of 4 or 5 (74, 75). Angiographic aneurysm occlusion after traditional coiling or WEB device deployment can be assessed by the Raymond–Roy occlusion scale (RROS) (51, 76). Adequate angiographic outcomes of aneurysm occlusion were defined as RROS class I (complete occlusion) or class 2 (near complete occlusion with a small residual neck). Aneurysm occlusion by FD deployment can be assessed by the O’Kelly Marotta (OKM) grading scale, which includes aneurysm occlusion from grade A-complete filling (>95%), grade B-incomplete filling (5–95%), grade C-neck remnant (<5%) and grade D-no filling (77).

#### 7.1.1 Traditional coiling

Traditional coiling for ICA bifurcation aneurysms can result in good outcomes in >90% of patients, as shown in Oishi’s et al. (44) report, Morales-Valero’s et al. (6) meta-analysis, Lee’s et al. (16) report and Ban’s et al. (5) report. An adequate angiographic occlusion at immediate post-EVT can be achieved in approximately 85% of ICA bifurcation aneurysms (5, 6, 9, 34, 44). However, a major limitation of traditional coiling is the recanalization due to hemodynamic stress (5, 6, 34, 44). In Ban’s et al. (5) report, after coiling, 21.2% of ICA bifurcation region aneurysms were recanalized during follow-up. A large aneurysm and low packing density are risk factors for recanalization (5, 37, 78). Additionally, superiorly projecting aneurysms may suffer recurrence compared with aneurysms without superiorly projecting aneurysms due to higher hemodynamic stress (17).

Recanalization can be divided into minor types, in which progressive filling limited to the neck of the aneurysm occurs, and major types, in which the aneurysmal sac is filled. Most recanalizations are the minor type (17). Retreatments were advocated for major recanalizations including coiling, FD deployment and clipping (51). According to the meta-analysis by Morales-Valero et al. (6), 14% of aneurysms required EVT retreatment.

#### 7.1.2 FD deployment

In highly selective ICA bifurcation region aneurysms, FD deployment can yield good outcomes. For instance, in Cagnazzo’s et al. (1) report, 20 ICA bifurcation aneurysms accounted for 47.6% of all 42 intracranial ICA aneurysms. After FD deployment, 88% of the aneurysms were completely or nearly completely occluded (OKM C and D) during a mean angiographic follow-up of 14 months, and the rate of morbidity was only 2.3%. In Mahmoud’s et al. (2) report of 7 patients with 8 ICA bifurcation aneurysms, all the aneurysms were cured by FD deployment. In addition, they also performed a literature review; a total of 38 patients harboring 42 aneurysms were identified, and FD deployment was associated with a >70% rate of complete or nearly complete aneurysm occlusion (2).

#### 7.1.3 WEB deployment

WEB device deployment for ICA bifurcation region aneurysms can yield good outcomes (79–83). Currently, there are no specialized studies that aim to treat ICA bifurcation aneurysms with the WEB device. The outcomes had to be extracted from those studies containing ICA bifurcation region aneurysms (79, 81, 84–87). In general, WEB deployment for ICA bifurcation region aneurysms can yield good outcomes in approximately 80% of cases, as shown in the studies of Pierot et al. (84), Dmytriw et al. (81), Cortese et al. (85), Pierot et al. (79), Lee et al. (86) and Hassankhani et al. (87).

Recently, more exciting results have been achieved. In Adeeb’s et al. (88) study, the outcomes of WEB device deployment for various intracranial bifurcation aneurysms (38 ICA bifurcation region aneurysms were included, accounting for 6.6% of 572 aneurysms) were compared; at the >1 year follow-up, a 96.7% rate of adequate occlusion was achieved for ICA bifurcation region aneurysms.

When using the WEB device to treat bifurcation aneurysms, the device may be compacted, resulting in aneurysm recurrence. In Dmytriw’s et al. (81) study, during at least 6 months of follow-up, minor compaction (<50%) occurred in 32.8% of bifurcation aneurysms, and major compaction was observed in 9.3% of the bifurcation aneurysms. Retreatment via stent-assisted coiling, FD or clipping may be necessary for 10% of aneurysms (61, 82, 83). Oversizing the WEB may result in slightly better angiographic treatment outcomes (89).

### 7.2 Complications

EVT for ICA bifurcation region aneurysms is associated with a risk of hemorrhagic and thromboembolic complications.

#### 7.2.1 Traditional coiling

In traditional coiling, due to unstable microcatheter positioning, intraoperative aneurysm rupture can occur in 3% of patients (6). Soft coils may be helpful. In addition, intraoperative thromboembolic complications can occur (6, 44). According to Ban et al. (5), complications due to thromboembolism occurred in 1.9% of patients. A high-metal coverage stent should be used cautiously to cover the MCA origin. Intraprocedural thrombus formation can be attempted by intra-arterial infusion of urokinase and/or tirofiban (5, 37, 44). Rarely, large ICA bifurcations can produce visual deficits via the mass effect of coiling because of the close proximity of the ICA (90). Therefore, coiling of large aneurysms should also be performed cautiously.

#### 7.2.2 FD deployment

When deploying FD to treat ICA bifurcation region aneurysms, intraprocedural and delayed thromboembolic complications can occur (Figure 8C). According to Cagnazzo et al. (1), 4.7% of ischemic events occurred, including basal ganglia infarction after coverage of the M1 perforators and transient acute in-stent thrombosis. In Mahmoud’s et al. (2) review, 7.9% of ischemic events were reported, including minor strokes and ganglionic infarctions. Intra-arterial infusion of urokinase and/or tirofiban can eliminate intraprocedural ischemic complications. In addition, delayed hemorrhage can rarely occur due to hemodynamic changes after FD deployment for ICA bifurcation region aneurysms (91). New generation of FDs with surface modifications that facilitate single antiplatelet therapy may have the potential to reduce the complication of delayed hemorrhage (92, 93).

#### 7.2.3 WEB deployment

WEB device deployment for ICA bifurcation region aneurysms can be associated with complications. The complications were thromboembolic events in 10% of the patients and hemorrhagic complications in 2% of the patients (81, 94–96). Intraprocedural thromboembolic events can be attempted by intra-arterial infusion of urokinase and/or tirofiban. When the WEB device protrudes into the parent artery, additional stent implantation may be used to avoid thromboembolic event (61, 97). However, most thromboembolic events are asymptomatic, and the major morbidity and mortality rates are reported to be <5%. Intraoperative bleeding during web deployment may often be disastrous, which halts successful WEB deployment (51). Coiling the ruptured bleb or choosing the appropriate size of WEB device was helpful for preventing intraoperative aneurysm rupture.

## 8 Summary

ICA bifurcation region aneurysms are uncommon. EVT can be a useful option for these aneurysms. Due to the complexity of these aneurysms and the variability of EVT techniques, EVT is challenging. Currently, traditional coiling is still a useful therapy for ICA bifurcation region aneurysms. In addition, the FD and Woven EndoBridge devices can be used in select cases. Some new devices are becoming promising.

## Author contributions

XL: Data curation, Writing – review & editing. YG: Writing – review & editing. KZ: Data curation, Writing – review & editing. JY: Conceptualization, Writing – original draft, Writing – review & editing.
